# Supplementary material for: Iron Dysregulation in Alzheimer’s Disease: LA-ICP-MS Bioimaging of the Distribution of Iron and Ferroportin in the CA1 Region of the Human Hippocampus
Source: Biomolecules. 2024 Mar 1;14(3):295. doi: 10.3390/biom14030295 (PMC10968591; doi:10.3390/biom14030295)
Supplement: Supplementary file 1 [file biomolecules-14-00295-s001.zip › biomolecules-2824389-supplementary.pdf]

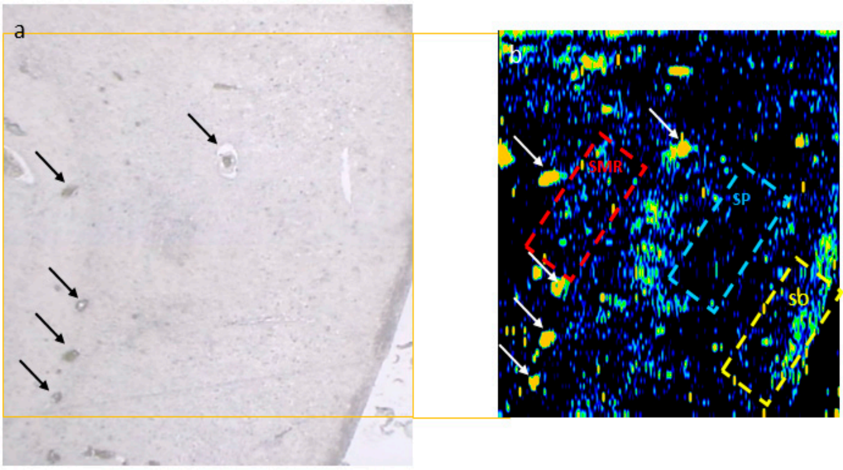

Figure S1. Evaluation of Fe distribution in CA1 sections using LA-ICP-MS (laser spot size diameter: 25 mm). (**a**) Transmission image from hippocampus CA1 region of AD brain (donor 6) taken with the laser camera. (**b**) Correspondent qualitative image of $^{56}Fe^{+}$ distribution. The arrows indicate what corresponds to large blood vessels in both images. The boxes indicate the areas where the data collection was carried out (red: stratum molecular–radial (SMR), blue: stratum pyramidale (SP) and yellow: stratum oriens (SO).
